# *Heliotropium hirsutissimum* from geothermal areas: evidence of thermal adaptation

**DOI:** 10.1007/s00709-025-02079-5

**Published:** 2025-06-05

**Authors:** Asiye Sezgin Muslu, Asim Kadıoğlu

**Affiliations:** Department of Biology, Faculty of Science, Karadeniz Technical University, 61080 Trabzon, Türkiye

**Keywords:** *Heliotropium hirsutissimum*, High temperature tolerance, Antioxidant capacity, Photosynthetic machinery, Chlorophyll fluorescence

## Abstract

*Heliotropium* L. genus belongs to the Boraginaceae family and is represented by approximately 250 species found in the temperate warm regions of the world, and there are 15 species of these species recorded in Türkiye. *Heliotropium hirsutissimum* Grauer grows in Bulgaria, Greece, N. Africa, Syria, and Türkiye. There is no record showing that *H. hirsutissimum* is a heat-tolerant plant. However, in our field studies, it was observed that *H. hirsutissimum*, which is also distributed in Hisaralan Thermal Springs of Sındırgı-Balıkesir, Türkiye, grows in the thermal area with extremely high soil temperature (57.6 °C (~ 60 °C)). It was thought that it would be useful to investigate the tolerance mechanism of the *H. hirsutissimum* plant to extremely high temperatures. For this purpose, the plant seeds were obtained from a geothermal area in the thermal spring. Growing plants were exposed to 20, 40, 60, and 80 ± 5 °C soil temperature gradually for 15 days under laboratory conditions. We measured the effect of high soil temperature on some morphological changes, relative water content, thiobarbituric acid reactive substances, cell membrane stability, and hydrogen peroxide analysis to determine stress levels on leaves and roots. Changes in osmolyte compounds, some antioxidant enzyme activities, ascorbate content, and chlorophyll fluorescence and photosynthetic gas exchange parameters were also determined. As a result of the study carried out to determine the stress level, it was observed that there was not much change and it was understood that the plant was tolerant to high soil temperature. In addition, there was a general increase in osmolytes accumulation, antioxidant enzyme activities, and ascorbate level. There was no significant difference in photosynthetic gas exchange and chlorophyll fluorescence parameters of plants grown at different soil temperatures. The high temperature did not negatively impact the photosynthetic yield of *H. hirsutissimum* because this plant was found to enhance its antioxidant capacity. The increase in antioxidant activity helped reduce oxidative damage and protect the photosynthetic mechanism under high temperature conditions, while the significant increase in the osmolyte level helped maintain the water status and cell membrane integrity of plants, thus enabling them to effectively withstand high soil temperatures.

## Introduction

Due to the detrimental effects of high temperature on plant development, global warming is predicted to have an overall negative effect on plant growth. Increasing extreme climatological threats, including very high temperatures, can cause huge losses in yield production and result in the spread of famine. The effects of high temperature stress on plants are of great importance due to the nutrition problem of the world population in the future. Because of this, it is seen to be crucial to comprehend how plants, which are the main providers of food, tolerate extreme temperatures, and to instill these traits, particularly in agricultural plants (Bita and Gerats 2013).

Over the course of evolution, plants have developed adaptive mechanisms to achieve a fine balance between growth and thermotolerance (Bahuguna and Jagadish 2015; Sedaghatmehr et al. 2019). In recent years, extensive genetic, physiological, molecular, and biochemical studies have shed light on how plants perceive stress signals and adapt to challenging environments. Thermo-sensing represents the initial step in the heat stress response (Jung et al. 2016, 2020). The cell membrane acts as the primary barrier against external stresses, with certain plasma membrane proteins potentially functioning as thermosensors that detect temperature fluctuations. Disruptions in the activity of these proteins can lead to significant physiological and morphological changes in plants (Zhu 2016; Ding et al. 2019; Vu et al. 2019; Zhang et al. 2022). In response to stress, plants commonly modify their growth and development to enhance survival. Deciphering the trade-offs between growth and heat stress adaptation is crucial for devising strategies to breed thermotolerant crops (Zhang et al. 2020, 2022).

Osmolytes such as proline, glycine-betaine, soluble sugars, sugar alcohols, and tertiary and quaternary ammonium compounds increase in response to high-temperature stress. Osmolyte accumulation is predicted to stabilize the membrane structure and increase protein stability (Sairam and Tyagi 2004; Wahid 2007). Accumulation of osmoprotectant, which provides osmotic regulation, is a significant adaptation mechanism in plants exposed to high temperatures (Allakhverdiev et al. 2008).

Plants’ high temperature resistance is enhanced by higher thermotolerance and delayed thermal damage (Horowitz 2001). Studies have shown that heat-stress injury can be prevented in heat-sensitive, cool-season plants by subjecting them to a heat-acclimation pretreatment; this was found to be partially due to the induction of antioxidative compounds, which helped to prevent the accumulation of reactive oxidant species (ROS) and membrane lipid peroxidation during heat stress (Xu et al. 2006). Heat stress modifies plants’ physiological, biochemical, and molecular responses. Photosynthetic activity is a temperature-dependent functional characteristic that influences plant metabolism and performance (Gratani et al. 2000). Studies have also shown that photosynthetic responses to heat stress exhibit synergistic tendencies involving internal CO_2_ concentration, net photosynthesis, and stomatal conductance; alterations in net photosynthesis were also connected with changes in stomatal control (Baker 2008; Dias et al. 2011). Therefore, it is vital to evaluate the variations in photosynthetic activity in plants under stress.

Furthermore, plants can adapt to high-temperature-induced oxidative stress by boosting the activities of enzyme involved in antioxidant systems. Plants have evolved an antioxidant defense system that includes both nonenzymatic and enzymatic elements in plant cells, reducing or even eliminating oxidative damage (Sairam et al. 2011; Signorelli et al. 2013). The activities of various antioxidant enzymes, such as catalase (CAT), ascorbate peroxidase (APX), superoxide dismutase (SOD), peroxidase (POX), and glutathione reductase (GR), have been observed to be influenced by temperature, as indicated by Hasanuzzaman et al. (2013). These enzymes exhibit their peak activations at distinct temperature thresholds. In a study involving six different *Lens culinaris* cultivars, it was noted that while CAT, APX, and SOD displayed an initial rise followed by a decrease at 50 °C, the activities of POX and GR decreased consistently across temperatures ranging from 20 to 50 °C (Chakraborty and Pradhan 2011). The research highlighted that thermotolerant plants tend to exhibit elevated levels of antioxidant enzymes and compounds due to the generation of ROS in chloroplast PS II, a major site for ROS accumulation, under conditions of high-temperature stress (Chakraborty and Pradhan 2011; Soliman et al. 2011; Suzuki et al. 2013).

Due to this decrease in carbon fixation, the amount of released oxygen increases and causes the formation of harmful ROS. The accumulation of ROS induces oxidative stress in plants. In conclusion, a heat-tolerant plant variety is typically characterized by enhanced membrane stability, improved heat retention capacity, and an elevated photosynthesis rate (Nagarajan et al. 2010). Furthermore, the ability of plants to sustain leaf gas exchange under high-temperature stress is closely linked to their tolerance to elevated temperatures.

Although the responses of plants to high temperatures have been widely investigated, the underlying mechanisms of thermotolerance remain not fully understood. It is imperative to analyze the strategies employed by thermophilic flora to combat high-temperature stress in order to comprehend the mechanisms underlying thermotolerance. Several thermophilic plant species thriving in warm environments have been documented in scientific literature. As reported by Tercek et al. (2003), *Agrostis scabra* (Willd.), a type of grass, has the ability to survive and flourish in soil temperatures reaching 45–50 °C. Moreover, research has revealed that *Campylopus pyriformis* (a moss species) can flourish at a soil temperature of 72 °C, while *Cyclosorus interruptus* (a fern species) and *Kunzea robusta* (a shrub variety) can thrive at 68 °C soil temperature (de Lange 2014; Smale and Fitzgerald 2015). Conversely, *Dichanthelium lanuginosum* (heat-tolerant flowering plants) found in the rhizosphere with temperatures exceeding 40 °C in Yellowstone National Park were documented, and alterations in the levels of sHSPs and HSP101 proteins in plants sampled from their natural habitat were identified (Stout and Al-Niemi 2002).

*Heliotropium* L. genus belongs to the Boraginaceae family and is represented by approximately 250 species found in the temperate warm regions of the world, and there are fifteen species of these species recorded in Türkiye. It is reported that among these species, the *Heliotropium thermophilum* plant lives in a geothermal area with temperatures of 55–65 °C in Aydın-Buharkent, while the *Heliotropium hirsutissimum* plant does not live in a thermal habitat but spreads in environments where the soil temperature is warmer (20–30 °C) (Tan et al. 2008). Although it is known that *H. thermophilum* is a thermophilic plant, no studies have been found showing that *H. hirsutissimum* is a temperature-tolerant plant in literatüre. However, in our field studies, it was observed that *H. hirsutissimum*, which is also distributed in Balıkesir-Sındırgı Hisaralan Thermal Springs, grows in the thermal area with extremely high soil temperature (57.6 °C (~ 60 °C)). However, the question of how *H. Hirsutissimum* adapts to an environment that poses significant stress for other plants remains unresolved. It was thought that it would be useful to investigate the tolerance mechanism of *H. hirsutissimum* plant to extremely high temperatures. Therefore, this study is considered important as it is the first study to investigate the extremely high temperature resistance mechanism of *H. hirsutissimum* under controlled conditions. The aim of this study was to investigate the high-temperature tolerance mechanism of *H. Hirsutissimum* and its relationship with photosynthetic capacity and antioxidant performance under natural conditions. For this reason, under laboratory conditions, to ascertain the thermotolerance mechanism of *H. hirsutissimum* grown at extremely high soil temperature conditions, measurements of growth parameters (shoot and root lengths), water potential, membrane damage, hydrogen peroxide level, and changes in some antioxidant enzyme activities were made in the leaves and roots. Then, photosynthetic gas exchange and chlorophyll fluorescence parameters were determined.

## Material and method

### Supply of plant seeds, growth and temperature applications

**Fig. 1:**
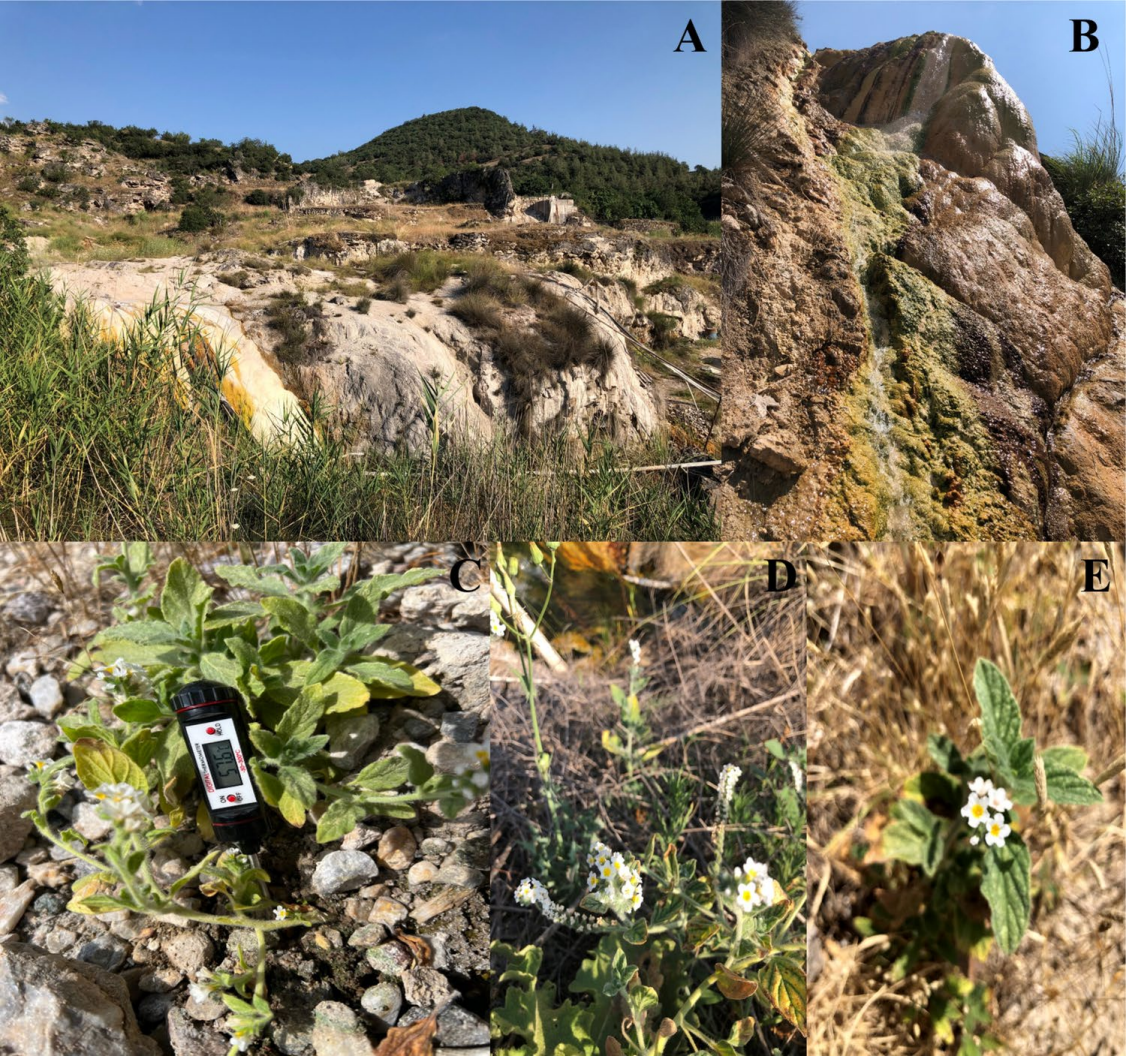
Natural habitat of *H. hirsutissimum* in Balıkesir–Sındırgı Hisaralan Thermal Springs (**A**-**B**), *H. hirsutissimum* plants in their natural habitat (**C**, **D**, and **E**)

The seeds of *Heliotropium hirsutissimum* were used as plant material in the current study. The seeds of plants were obtained from Balıkesir-Sındırgı Hisaralan Thermal Springs (57.6 °C, 39,26,936 ^ο^K, 28,31706 ^ο^D) on July 24 th, 2021 (Fig. 1). Plant seeds were sown in pots containing soil in the plant growth room (23 ± 2 °C, 50% humidity, light intensity 400 µmol m^−2^ s^−1^, 16-h light/8-h dark period). Soil waters were checked regularly. Based on the experiments, in order to prevent plant deaths and to carry out the studies in a healthy way, the seeds that germinated at 20 ± 5 °C were gradually exposed to temperatures under controlled conditions in the laboratory. Temperature applications were achieved by heating the soil in heat units adjusted to the desired temperature. The grouping was formed in 4 different ways for the plant species as groups that continue to grow at a soil temperature of 20 ± 5 °C, gradually increased stepwise, 20 °C every 15 days to 40, 60, and finally 80 °C. The temperature of the soil was periodically monitored by a digital thermometer at a depth of 5 cm. After the soil temperature reached 80 ± 5 °C, the plants were kept in a growth chamber for 15 days at each temperature. The soil temperature of the control group was kept at 20 ± 5 °C for 75 days (Sezgin Muslu and Kadıoğlu 2021a). All plants were regularly watered every day and harvested after 75 days. Each temperature treatment group was prepared in 4 sets, with each set holding 6 individual plants. The leaves and roots of the plants were used for RWC, TBARS, H_2_O_2_, and antioxidant enzymes analysis. The leaves of the plants were used for chlorophyll fluorescence and gas exchange parameters.

### Determination of leaf surface temperatures

Leaf surface temperatures were obtained with the infrared thermography method. Fluke TiS 60 + thermal camera was used in the measurements, and radiometric leaf surface temperature distribution was recorded with it. The obtained data was then processed on the computer via the camera’s software.

### Determination of shoot and root lengths

Plants belonging to each experimental group were harvested at the end of 10 weeks (75 days) and the average root and shoot length per plant was measured using a scala, recorded, and photographed.

### Determination of leaf and root relative water content (RWC)

The data were recorded by measuring the fresh leaf and root weight of the *H. hirsutissimum* plant. Then, the turgid weights were calculated by keeping the samples in tubes containing distilled water for 16 h. For the dry weight calculation, the turgid weights of the samples were determined and recorded and calculated after they were kept in an oven at 80 °C for 48 h (Castillo 1996).

### Determination of thiobarbituric acid reactive substances (TBARS) content

The level of TBARS of the *H. hirsutissimum* plant was measured following the method of Heath and Packer (1968). Samples (0.1 g) were homogenized in 1.8 mL of 0.1% trichloroacetic acid (TCA). The homogenate was centrifuged at 15,000 × g for 10 min. After adding 4 mL of 0.5% thiobarbituric acid prepared in 20% TCA to 1 mL of the supernatant, the absorbance of the supernatant was recorded at 532 nm. The reading for non-specific absorption at 600 nm was subtracted from the calculation. TBARS concentration was calculated by substituting the obtained result in the formula (A = E.c.l).

### Cell membrane stability (CMS)

CMS was assessed using a conductometric method. Leaf and root discs were collected from plants exposed to 20 ± 5 °C, 40 ± 5 °C, 60 ± 5 °C, and 80 ± 5 °C treatments. The discs were first rinsed with 20 mL of deionized water and subsequently incubated for 24 h. Following incubation, the initial electrolyte leakage was measured using a conductivity meter (Delta OHM, HD2306). These measurements were designated as C1 for the 20 ± 5 °C treatment and T1 for the higher temperature treatments (40 ± 5 °C, 60 ± 5 °C, and 80 ± 5 °C). Afterward, the samples were subjected to a heat treatment in a water bath for 15 min to completely kill the tissues, and conductivity was measured again. These final measurements were recorded as C2 for the 20 ± 5 °C treatment and T2 for the higher temperature treatments. CMS values were then calculated according to the method described by Tripathy et al. (2000):

$$\text{CMS}\%=1-(\text{T}1/\text{T}2)/(\text{C}2/\text{C}1)\times 100$$

### Determination of hydrogen peroxide (H_2_O_2_) content

Hydrogen peroxide determination of *H. hirsutissimum* plant was done according to Velikova et al. (2000). For this, the extract obtained from the plant samples (0.1 g) crushed with activated charcoal in 1.8 mL of 0.1% TCA was centrifuged; 1 mL was taken from the supernatant, 10 mM potassium phosphate buffer and 1 M KI were added to it, and its absorbance was read at 390 nm.

### Determination of changes in osmolytes compounds

For the proline content, fresh samples (0.1 g) were homogenized with ethanol: water (40:60 v/v). The mixture was left overnight at 4 °C, and then centrifuged at 15,000 × g (10 min). The reaction mix (ninhydrin 1% (w/v) in acetic acid 60% (v/v), ethanol 20% (v/v)) was added to 1 mL of supernatant. The mixture was then placed in tubes at 100 °C for 1 h in a water bath. To the cooled mixture, measured at 520 nm using a spectrophotometer (Carillo and Gibon 2011).

For the determination of total soluble sugar content, 0.2 g of dried leaf and root tissues were homogenized with 5 mL of 70% ethanol. The homogenate was then heated at 80 °C for 3 min and subsequently centrifuged at 10,000 × g for 5 min. From the resulting supernatant, 100 µL was mixed with 900 µL of distilled water. To this mixture, 1 mL of 5% phenol and 5 mL of 96% sulfuric acid were added. The absorbance of the final solution was measured at 490 nm following the method described by Dubois et al. (1956).

For the determination of glycine betaine content, 0.1 g of finely ground dry plant materials (leaves and roots) were extracted by mechanically shaking with 20 mL of deionized water for 48 h at 25 °C. After filtration, the extract was diluted 1:1 with 2 N sulfuric acid. Subsequently, 0.2 mL of potassium iodide–iodine reagent was added, and the mixture was centrifuged at 10,000 × g for 15 min. Then, 1 mL of the supernatant was mixed with 9 mL of 1,2-dichloroethane, and the absorbance was recorded at 365 nm, following the method described by Greive and Grattan (1983).

### Determination of changes in antioxidant capacity

Plant samples (0.1 g) were weighed and pulverized in liquid nitrogen. Then, 1.8 mL of extraction buffer (50 mM K_2_HPO_4_, 1 mM EDTA pH 7.0, 1% PVPP) was extracted. The extract was centrifuged at 16,000 × g for 20 min at 4 °C, and the supernatant was used for the determination of enzyme activity. 5 mM ascorbic acid was added to the buffer for ascorbate peroxidase (Demiralay et al. 2013; Terzi et al. 2018).

Catalase (CAT) activity was determined according to the method of Aebi (1983). Enzyme activity was determined by measuring the 1 mM reaction mixture containing 50 mM potassium phosphate buffer (pH 7.0), 30 mM H_2_O_2_, and 20 µL enzyme extract at 240 nm for 5 min. The activity of ascorbate peroxidase (APX) was determined by the decrease in absorbance at 290 nm (Nakano and Asada 1981). Enzyme activity was determined by measuring a 1 mL reaction mixture containing 50 mM potassium phosphate buffer (pH 7.0), 250 µM ascorbate (ASC), 5 mM H_2_O_2_, and 20 µL enzyme extract. The absorbance values of APX were determined at 290 nm. The method described by Urbanek et al. (1991) was used to determine the guaiacol peroxidase (GPX) activity of the leaves. Enzyme activity was determined by measuring the 2 mM reaction mixture with 100 mM potassium phosphate buffer (pH 7.0), 0.1 mM EDTA, 5 mM guaiacol, 15 mM H_2_O_2_, and 50 µL enzyme extract at 470 nm for 1 min. To calculate enzyme activities, protein concentration was determined spectrophotometrically using the Bradford method (1976).

The ascorbate content was determined by using Liso et al. (1984). Fresh leaves (0.1 g) were homogenized with 1.8 mL of 5% (w/v) m-phosphoric acid. Extract 4 min at 10,000 × g centrifuged. 70 µL of sample, 3 mL containing 0.1 M citrate-0.2 M phosphate buffer (pH 6.2) was added to the reaction medium. The initial absorbance was read at 265 nm; the concentration of ascorbate was determined by reading the decrease after 5 min of addition of two units of ascorbate oxidase to the reaction medium. The ascorbate concentration was calculated as microgram ascorbate per fresh weight.

### Determination of photosynthetic gas exchange and chlorophyll fluorescence parameters

Stomatal conductivity, photosynthesis rate, and transpiration rate measurements were performed with a portable system, the TPS-2 Photosynthesis System (PP Systems, Hertfordshire, UK). Measurements were made in laboratory conditions by selecting 6 healthy leaves from at least 4 plants. The air flow rate to the cuvette was set at 300 mL min^−1^.

Chlorophyll fluorescence measurements were performed by a fluorometer (OS1-FL) (Nar et al. 2009). Measurements were taken from 6 different leaves exposed to the dark for 20 min. Minimum chlorophyll fluorescence (F_0_) and maximum chlorophyll fluorescence (F_m_) were obtained by applying λ690 white light (8000 µmol m^–2^ s^–1^), which saturates PS2, for 0.8 s. After dark measurements, steady-state chlorophyll fluorescence (Fs) was obtained by applying actinic light (5.5 W halogen lamp, ML S990, Micron, Tokyo, Japan) to the leaves. Saturating white light (8000 µmol m^–2^ s^–1^) was applied for 0.8 s to determine the maximum chlorophyll fluorescence (Fm’) in the light. The fluorescence parameters were calculated using the following formulas (van Kooten and Snel 1990): the maximum quantum yield of PSII photochemistry, F_v_/F_m_ = (F_m_—F_0_)/F_m_; photochemical quenching of variable chlorophyll fluorescence, qP = (F’_m_ -F_s_)/(F’_m_-F’_0_); and nonphotochemical chlorophyll fluorescence quenching, NPQ = (F_m_-F’_m_)/F_m_. The effective quantum yield of ФPSII = (F_m_’-F_s_)/F_m_’) of Genty et al. (1989), and the electron transfer rate (ETR) of Nar et al. (2009) were also determined.

### Statistical analysis

Statistical analysis was carried out using one-way ANOVA variance analysis tests (Duncan Multiple Comparison Test) with the Statistical Package for Social Sciences. *P* < 0.05 was considered statistically significant. Pearson’s correlation analysis was performed to reveal the relationship between all the parameters. Variables were further subjected to principal component analysis (PCA) using the MVSP computer program.

## Results

### Leaf surface temperatures

**Fig. 2:**
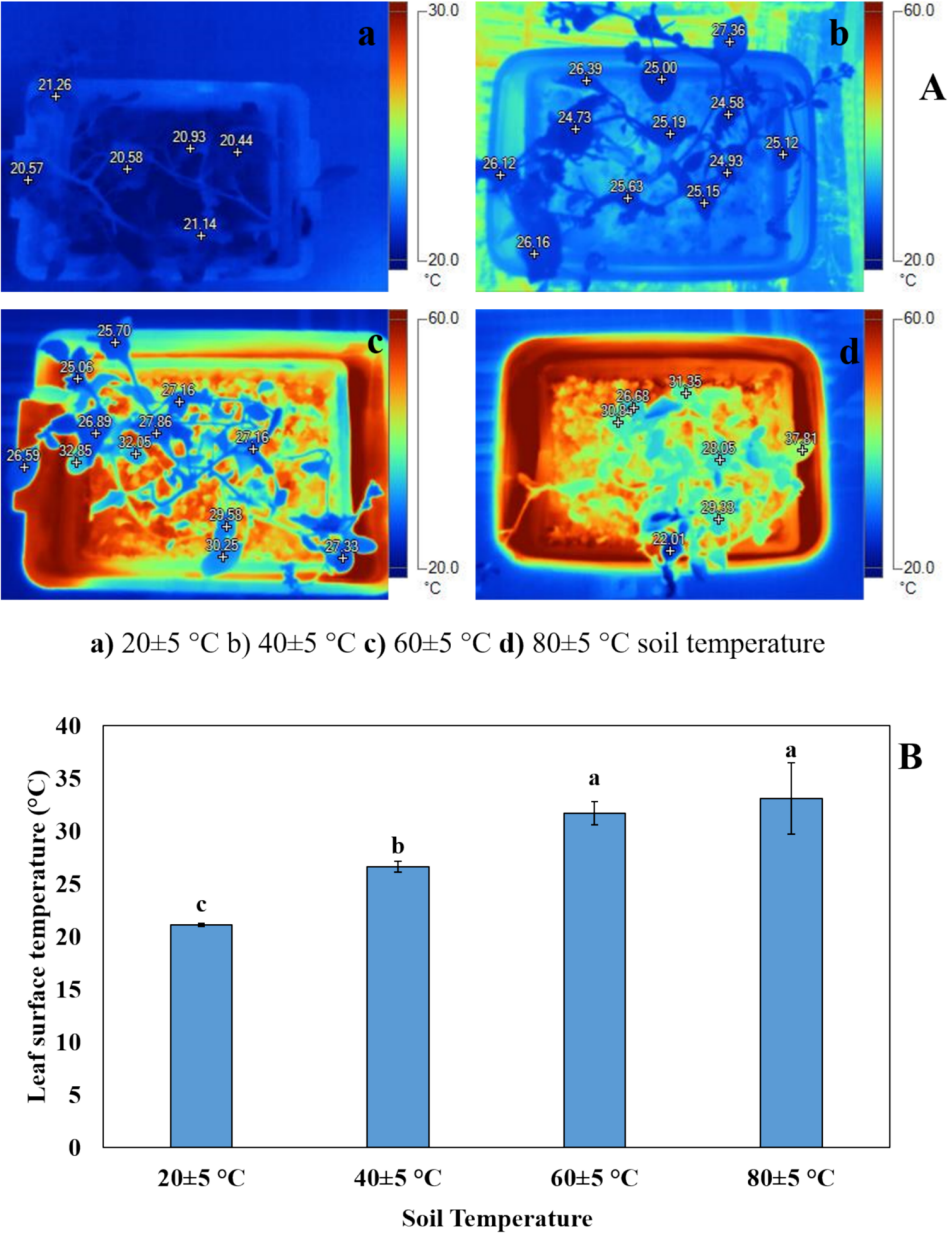
Radiometric distribution of leaf surface temperatures (**A**), leaf surface temperature (**B**). Bars, s.d. Different lowercase letters indicate significant differences between treatments at *P* < 0.05

Leaf surface temperatures of plants grown at different soil temperatures were shown as radiometric distribution with a thermal camera (Fig. 2A). It was found that leaf surface temperatures increased with increasing soil temperature. No statistically significant difference was observed between the leaf surface temperatures of plants grown at 60 ± 5 °C (31.72 ± 1.09 °C) and 80 ± 5 °C (33.08 ± 3.39 °C) soil temperatures. Additionally, it was found that the leaf surface temperature of plants grown at 40 ± 5 °C (26.64 ± 0.52 °C) soil temperature was higher than that of plants grown at 20 ± 5 °C (21.11 ± 0.14 °C) (Fig. 2B).

### Changes in plant morphology

**Fig. 3:**
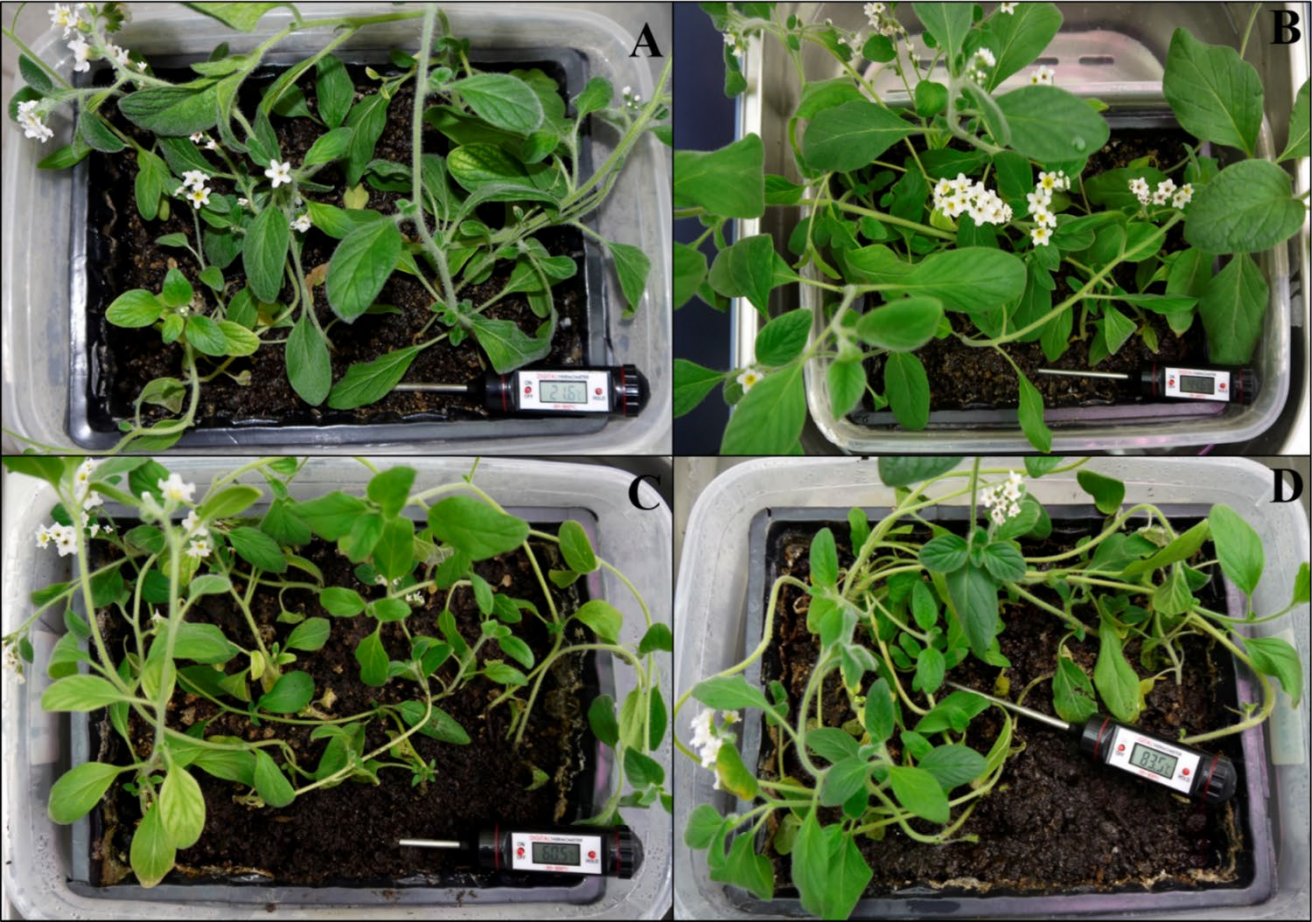
75-day-old plants grown at different temperatures (20 ± 5 °C (**A**), 40 ± 5 °C (**B**), 60 ± 5 °C (**C**), and 80 ± 5 °C (**D**)) under laboratory conditions

Morphological changes in *H. hirsutissimum* plants at the end of 10 weeks, where different temperatures were applied for 2 weeks by gradually increasing the temperature in the soil (20, 40, 60, and 80 ± 5 °C), were presented in Fig. 3. The best growth was seen in plants grown at 40 ± 5 °C. No significant difference was seen when plants grown at 60 ± 5 °C and 80 ± 5 °C were compared. Finally, it was found that plants grown at 60 ± 5 °C and 80 ± 5 °C had slower morphological development compared to other temperature treatments. In addition, no deformation in the plants, changes such as excessive senescence and pigment loss in the plant leaves due to increasing temperature (including 80 ± 5 °C) were observed.

**Fig. 4:**
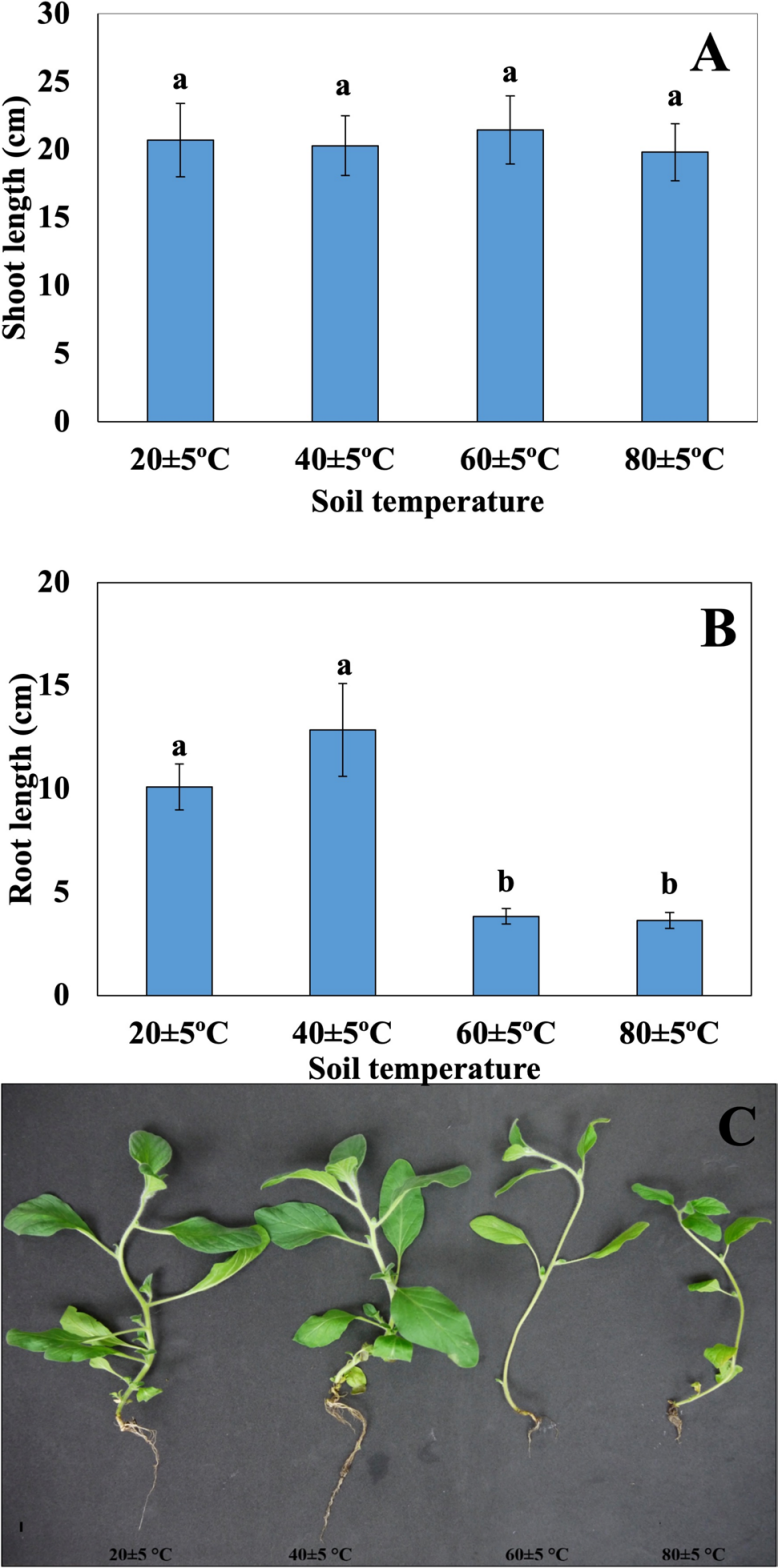
Shoot lengths of plants at different soil temperatures (**A**), root lengths of plants at different soil temperatures (**B**), photograph of shoot and root lengths of plants at different soil temperatures (**C**). Bars, s.d. Different lowercase letters indicate significant differences between treatments at *P* < 0.05

There was no statistically significant difference in shoot lengths of plants grown at different soil temperatures. When the root lengths were examined, a decrease was observed in the root lengths with increasing temperatures. At the same time, no statistically significant difference was observed between the root lengths of plants grown at 20 ± 5 °C and 40 ± 5 °C soil temperatures and the root lengths of plants grown at 60 ± 5 °C and 80 ± 5 °C soil temperatures (Fig. 4).

### Leaf and root RWC

**Fig. 5:**
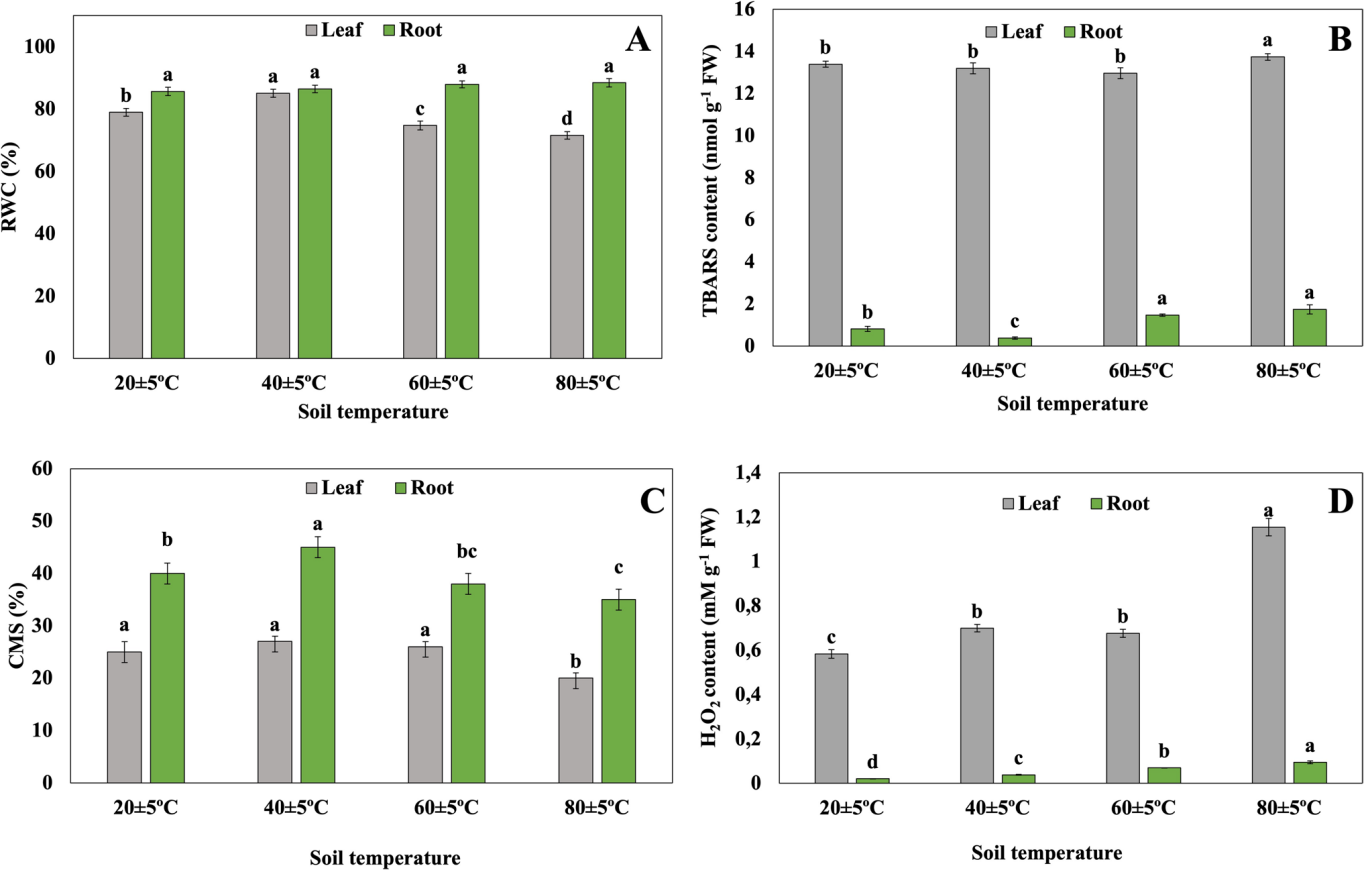
Changes of RWC (**A**), TBARS content (**B**)**,** CMS (**C**), and H_2_O_2_ content (**D**) in leaves and roots of plants grown at different temperatures. Bars, s.d. Different lowercase letters indicate significant differences between treatments at *P* < 0.05

Compared to 20 ± 5 °C soil temperature, the relative water content of leaves of plants grown at 40 ± 5 °C soil temperature increased, while the relative water content of leaves of plants grown at 60 ± 5 °C and 80 ± 5 °C soil temperature gradually decreased. No statistically significant difference was observed between the relative water contents of roots of plants grown at different soil temperatures (Fig. 5A).

### TBARS content

While there was no statistically significant difference in the TBARS content in the leaves of plants grown at different soil temperatures, it increased in plants at 80 ± 5 °C soil temperature. Compared to 20 ± 5 °C soil temperature, it was found that the TBARS content in the roots of plants grown at 40 ± 5 °C soil temperature decreased, while the TBARS content in the roots of plants grown at 60 ± 5 °C and 80 ± 5 °C soil temperature increased. At the same time, no difference was found in terms of TBARS content in the roots of plants at 60 ± 5 °C and 80 ± 5 °C (Fig. 5B).

#### CMS

In terms of CMS in leaves, there was no statistically significant difference in CMS of plant leaves grown at 20, 40, and 60 °C soil temperatures, while a slight decrease was found in those at 80 ± 5 °C. CMS in roots was higher compared to leaves, and CMS in roots of plants grown at 40 ± 5 °C soil temperatures increased when compared to other temperature groups, while no statistically significant difference was found between the CMS of other groups (Fig. 5C).

### H_2_O_2_ content

The H_2_O_2_ content in plant leaves increased as the soil temperature increased from 20 ± 5 °C to 80 ± 5 °C. At the same time, no statistically significant difference was found between the H_2_O_2_ content in the leaves of plants at soil temperatures of 40 ± 5 °C and 60 ± 5 °C. Compared to 20 ± 5 °C soil temperature, it was found that the H_2_O_2_ content in the roots of plants grown at 40 ± 5 °C soil temperature decreased, while the H_2_O_2_ content in the roots of plants grown at 60 ± 5 °C and 80 ± 5 °C soil temperatures increased (Fig. 5D).

### Changes in osmolytes compounds

**Fig. 6:**
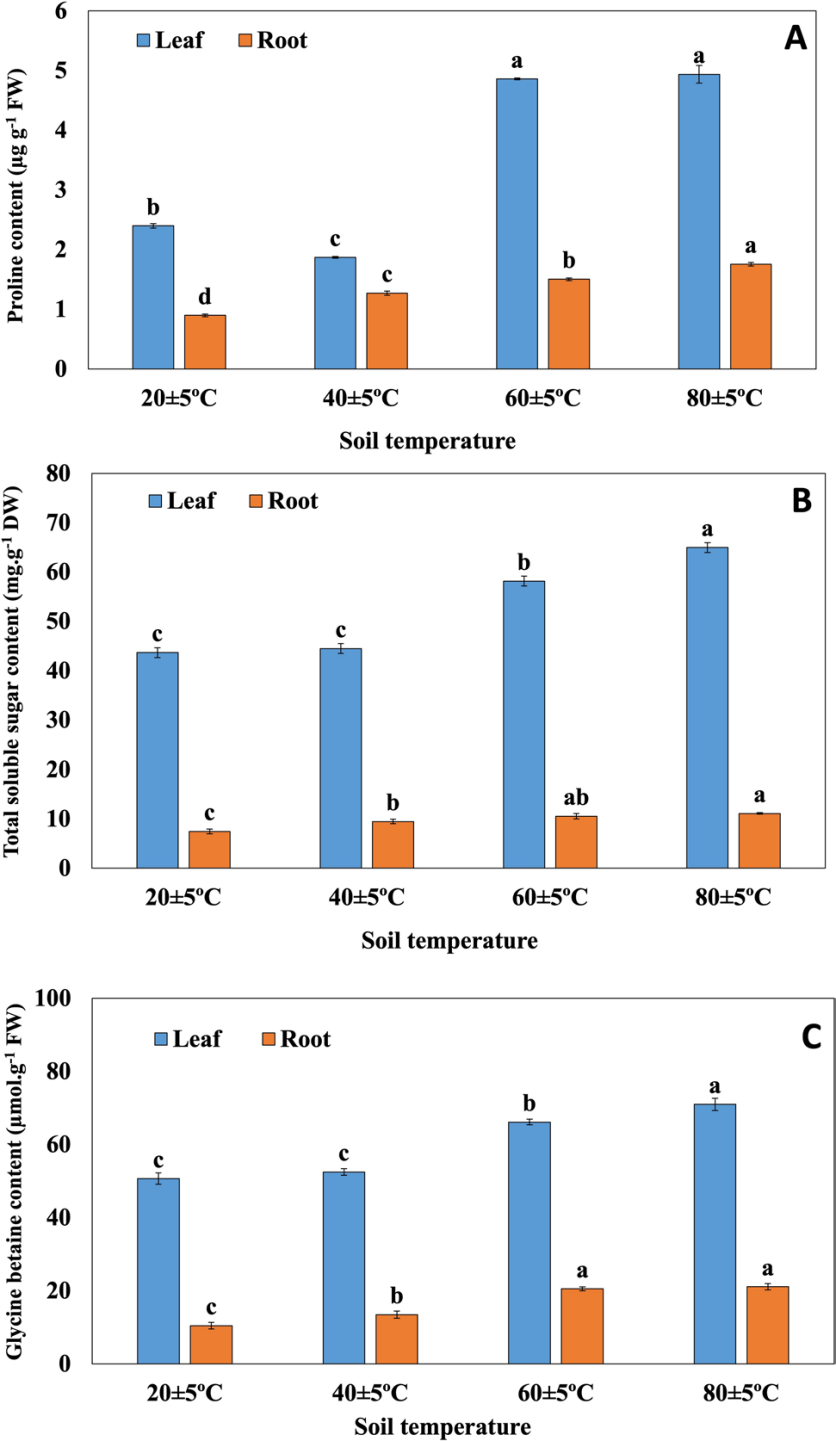
Changes of proline content (**A**), total soluble sugar content (**B**), glycine betaine content (**C**) in leaves and roots of plants grown at different temperatures. Bars, s.d. Different lowercase letters indicate significant differences between treatments at *P* < 0.05

While proline content decreased in the leaves of plants grown at 40 ± 5 °C soil temperature compared to 20 ± 5 °C soil temperature, proline content increased significantly in the leaves of plants grown at 60 ± 5 °C and 80 ± 5 °C. The proline content in the roots increased with rising temperature (Fig. 6A). Total soluble sugar content in leaves and roots of *H. hirsutissimum* plants increased with rising temperature (Fig. 6B). No statistically significant difference was observed between the glycine betaine content of the leaves of plants grown at 20 ± 5nd 40 ± 5oil temperatures and the glycine betaine content of the roots of plants grown at 60 ± 5 °C and 80 ± 5 °C soil temperatures. Moreover, glycine betaine content increased significantly in the leaves of plants grown at 60 ± 5 °C and 80 ± 5 °C, compared to 20 ± 5 °C soil temperature. It was also increased in the roots of plants grown at 40 ± 5 °C, 60 ± 5 °C, and 80 ± 5 °C (Fig. 6C).

### Changes in antioxidant capacity

**Fig. 7:**
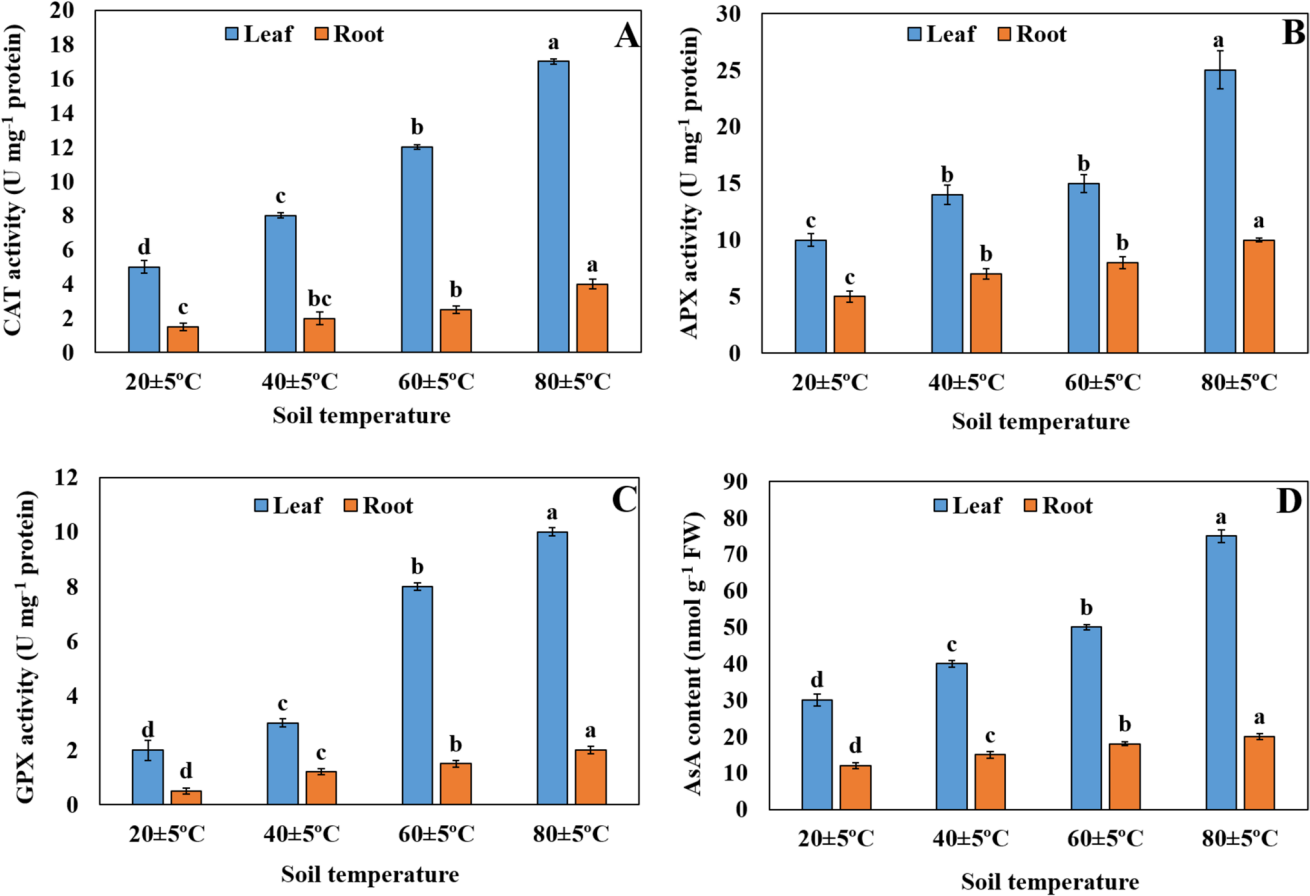
Changes of CAT activity (**A**), APX activity (**B**), GPX activity (**C**), and AsA content (**D**) in leaves and roots of plants grown at different temperatures. Bars, s.d. Different lowercase letters indicate significant differences between treatments at *P* < 0.05

CAT activities of leaves and roots of plants were found to increase with rising soil temperature compared to plants growing at 20 ± 5 °C. It was also noted that this increase was greater in leaves (Fig. 7A). APX activity in plant leaves and roots increased as the soil temperature increased from 20 ± 5 °C to 80 ± 5 °C. At the same time, no statistically significant difference was found between the APX activity in the leaves and roots of plants at soil temperatures of 40 ± 5 °C and 60 ± 5 °C (Fig. 7B). GPX activity of plants was found to increase with rising soil temperature compared to plants growing at 20 ± 5 °C. Moreover, it was determined that the increment in the GPX activity was greater in leaves (Fig. 7C). AsA content in plant leaves and roots of plants significantly increased as the soil temperature increased from 20 ± 5 °C to 80 ± 5 °C (Fig. 7D).

### Changes in photosynthetic gas exchange and chlorophyll fluorescence parameters

**Fig. 8:**
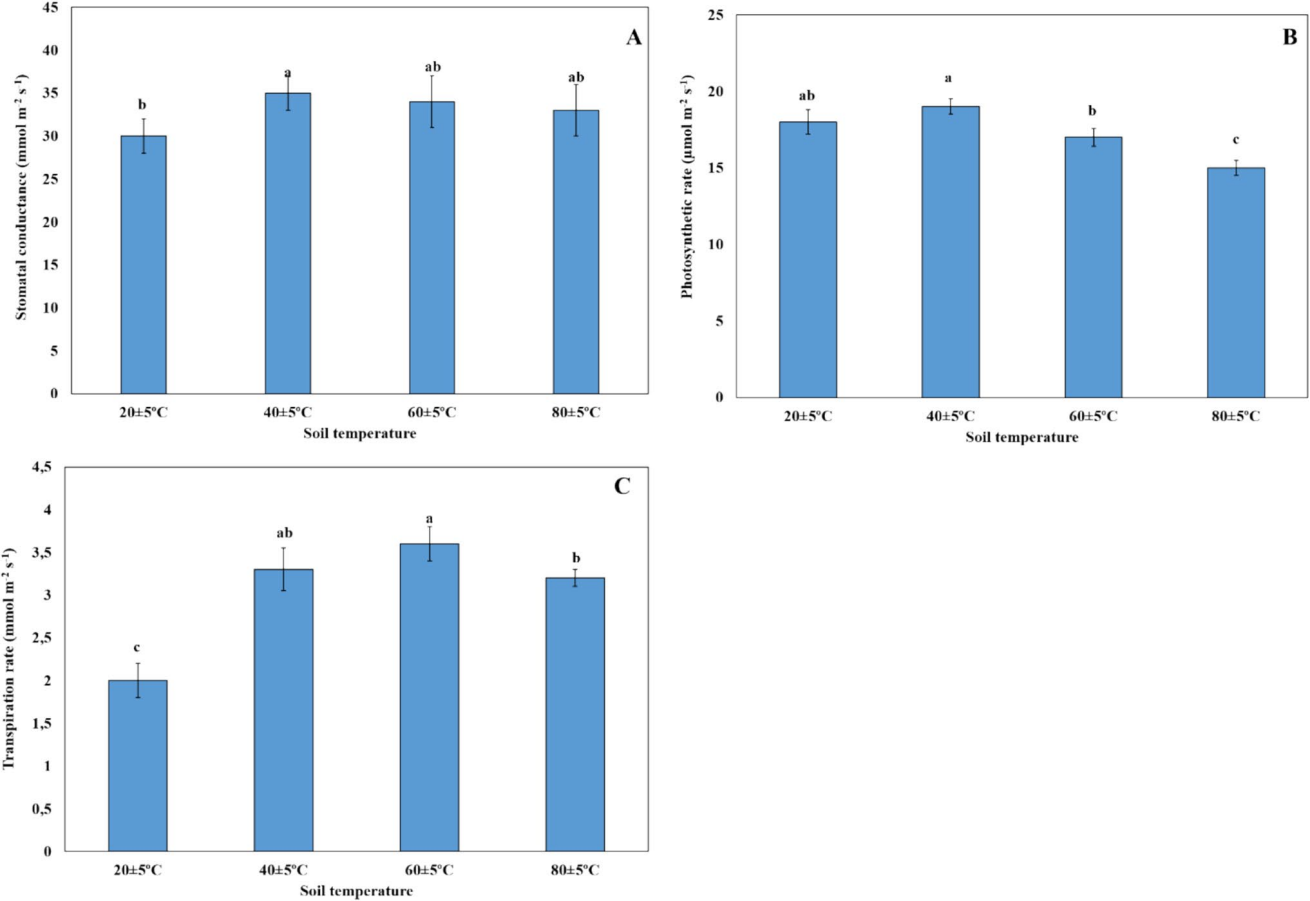
Changes of gas exchange parameters: Stomatal conductance (**A**), photosynthetic rate (**B**), and transpiration rate (**C**) of plants grown at different temperatures. Bars, s.d. Different lowercase letters indicate significant differences between treatments at *P* < 0.05

The stomatal conductance of plants living at 40 ± 5 °C was higher than that of plants living at 20 ± 5 °C. There was no statistically significant difference in stomatal conductance between plants grown at 60 ± 5 °C and 80 ± 5 °C compared to those grown at 20 ± 5 °C and 40 ± 5 °C (Fig. 8A). There was no statistically significant difference between 20 ± 5 °C and 40 ± 5 °C, and between 20 ± 5 °C and 60 ± 5 °C in terms of photosynthetic rate of plants. However, the photosynthetic rate of plants growing at 80 ± 5 °C decreased compared to those grown at other temperatures (Fig. 8B). Compared to plants groving at 20 ± 5 °C, transpiration rate was found to increase with rising soil temperature. Additionally, it was found that the transpiration rate of plants grown at 40 ± 5 °C did not change statistically when compared to plants grown at 60 ± 5 °C and 80 ± 5 °C. It was also found to decrease in the transpiration rate of plants growing at 80 ± 5 °C compared to those grown at 60 ± 5 °C (Fig. 8C).

**Fig. 9:**
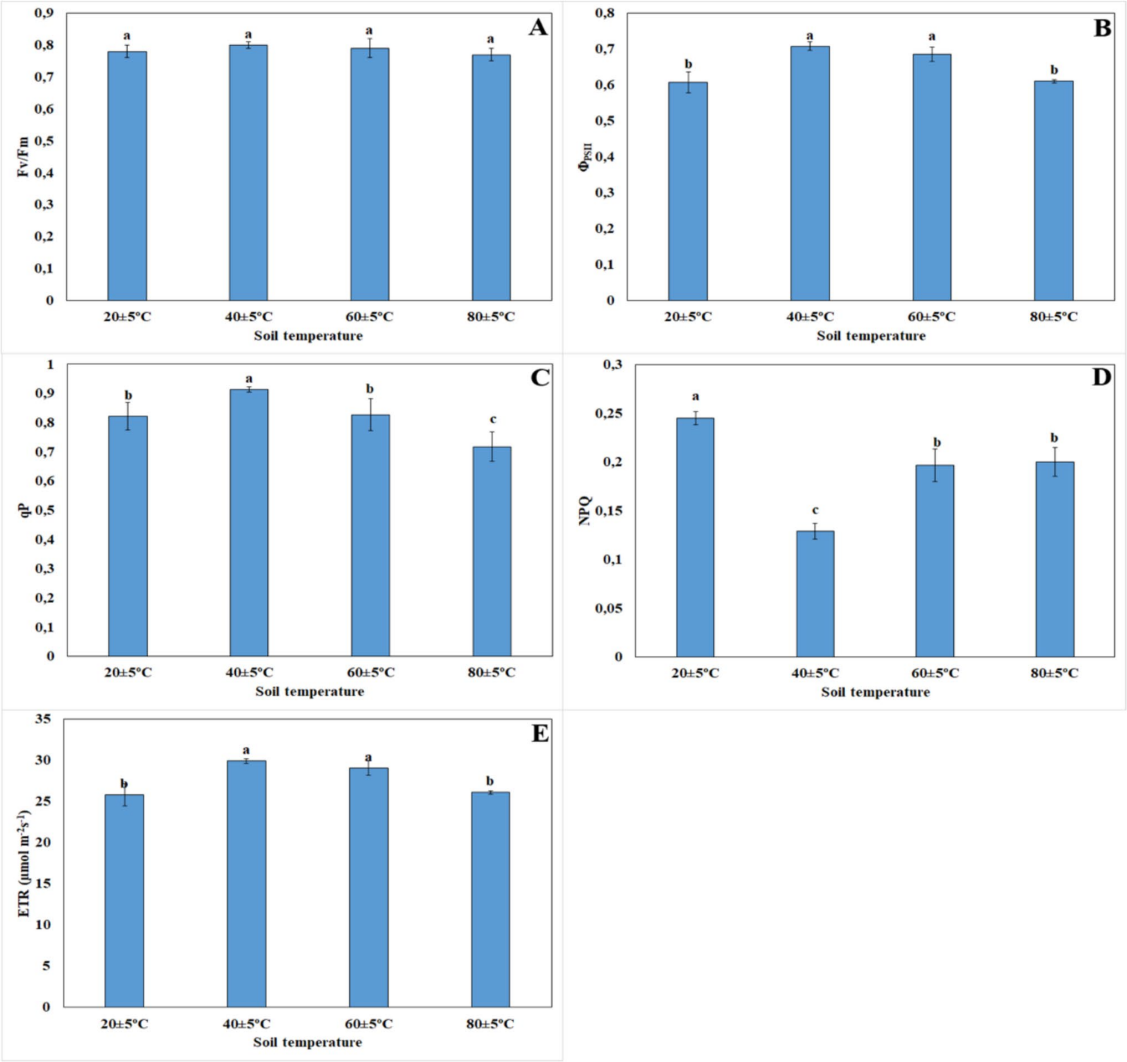
Changes of chlorophyll fluorescence parameters: Fv/Fm (**A**), Q_PSII_ (**B**), qP (**C**), NPQ (**D**), ETR (**E**) of plants grown at different temperatures. Bars, s.d. Different lowercase letters indicate significant differences between treatments at *P* < 0.05

There was no statistically significant difference among plants grown at 20 ± 5 °C, 40 ± 5 °C, 60 ± 5 °C, and 80 ± 5 °C in terms of the Fv/Fm ratio (Fig. 9A). The photochemical yield of PSII in plants living at 40 ± 5 °C and 60 ± 5 °C was higher compared to that of plants living at 20 ± 5 °C. Moreover, there was no statistically significant difference between plants grown at 20 ± 5 °C and 80 ± 5 °C in terms of the photochemical yield of PSII (Fig. 9B). The qP in plants living at 40 ± 5 °C increased, while the qP in plants living at 80 ± 5 °C decreased compared to that in plants at 20 ± 5 °C. Additionally, there was no statistically significant difference between plants grown at 20 ± 5 °C and 60 ± 5 °C (Fig. 9C). As shown in the data in Fig. 9D, the amount of NPQ in plants living at high soil temperatures was lower than that in plants at 20 ± 5 °C. The ETR of plants at 60 ± 5 °C and 80 ± 5 °C was higher than that of plants at 20 ± 5 °C. There was also no statistically significant difference between plants grown at 20 ± 5 °C and 80 ± 5 °C in terms of the ETR (Fig. 9E).

### Correlation of all the parameters in the leaves and roots of *H. hirsutissimum* grown at different soil temperatures (20 ± 5 °C, 40 ± 5 °C, 60 ± 5 °C, and 80 ± 5 °C)

The association between all parameters of the leaves of *H. hirsutissimum* at 20 ± 5 °C, 40 ± 5 °C, 60 ± 5 °C, and 80 ± 5 °C soil temperatures was depicted in a Pearson correlation table (Table 1). The leaf RWC indicated correlation with H_2_O_2_ level (*r* = − 0.601*), CAT (*r* = − 0.742**), APX (*r* = − 0.618*), GPX (*r* = 0.825**) activities, and AsA (*r* = − 0.720**) level. The leaf RWC also showed a strong negative correlation with proline (*r* = − 0.904**), total soluble sugar (*r* = − 0.851**), and glycine betaine (*r* = − 0.823**) content. A significant correlation (*p* < *0.01*) was obtained between H_2_O_2_ level and antioxidant parameters. H_2_O_2_ level indicated a strong positive correlation with CAT (*r* = 0.892**), APX (*r* = 0.982**), GPX (0.782**) activities, and AsA (*r* = 0.951**) level. H_2_O_2_ content also showed negative correlation with CMS (*r* = − 0.801**). The stomatal conductance exhibited a positive correlation with the transpiration rate (*r* = 0.765**), Fv/Fm (*r* = 0.823**), QPSII (*r* = 0.721**) and with the ETR (*r* = 0.735**) (*p* < *0.01*).

**Table 1:** Pearson’s correlation analysis of all the parameters in the leaves of *H. hirsutissimum* grown at different soil temperatures (20 ± 5 °C, 40 ± 5 °C, 60 ± 5 °C, and 80 ± 5 °C). Pro: proline, TS: Total sugars, GB: Glycine betaine, SC: Stomatal conductance, PR: Photosynthetic rate. TR: Transpiration rate

	RWC	TBARS	CMS	H_2_O_2_	Pro	TS	GB	CAT	APX	GPX	AsA	SC	PR	TR	Fv/Fm	QPSII	qP	NPQ	ETR
RWC	**1**																		
TBARS	− 0.239	**1**																	
CMS	0.775**	− 0.534	**1**																
H_2_O_2_	− 0.601*	0.684*	− 0.801**	**1**															
Pro	− 0.904**	0.144	− 0.548	0.604*	**1**														
TS	− 0.851**	0.339	− 0.654*	0.807**	0.954**	**1**													
GB	− 0.823**	0.283	− 0.584*	0.771**	0.959**	0.995**	**1**												
CAT	− 0.742**	0.370	− 0.674*	0.892**	0.856**	0.966**	0.960**	**1**											
APX	− 0.618*	0.602*	− 0.725**	0.982**	0.693*	0.876**	0.854**	0.948**	**1**										
GPX	− 0.825**	0.260	− 0.610*	0.782**	0.952**	0.994***	0.997**	0.972**	0.861**	**1**									
AsA	− 0.720**	0.491	− 0.736**	0.951**	0.797**	0.939**	0.922**	0.998**	0.983**	0.933**	**1**								
SC	0.286	0.147	0.400	0.195	0.115	0.225	0.278	0.283	0.317	0.247	0.257	**1**							
PR	0.944**	− 0.338	0.918**	− 0.770**	− 0.823*	− 0.855**	− 0.812**	− 0.823**	− 0.760**	− 0.832**	− 0.829**	0.282	**1**						
TR	− 0.108	− 0.156	0.121	0.349	0.492	0.553	0.613*	0.621*	0.499	0.619*	0.537	0.765**	− 0.133	**1**					
Fv/Fm	0.588*	0.021	0.738**	− 0.279	− 0.268	− 0.244	− 0.188	− 0.240	− 0.192	− 0.232	− 0.261	0.823**	0.681*	0.369	**1**				
QPSII	0.586*	− 0.494	0.767**	− 0.337	− 0.212	− 0.209	− 0.127	− 0.127	− 0.213	− 0.127	− 0.211	0.721**	0.609*	0.684*	0.714**	**1**			
qP	0.888*	− 0.348	0.930**	− 0.636*	− 0.652*	− 0.664*	− 0.606*	− 0.621*	− 0.582*	− 0.629*	− 0.646*	0.542	0.942**	0.144	0.841***	0.781**	**1**		
NPQ	− 0.476	0.348	− 0.250	− 0.054	0.269	0.126	0.089	− 0.090	− 0.096	0.043	− 0.066	− 0.411	− 0.268	− 0.576*	− 0.176	− 0.694*	− 0.387	**1**	
ETR	0.556	− 0.478	0.742**	− 0.299	0.177	− 0.171	− 0.087	− 0.086	− 0.174	− 0.087	− 0.169	0.735**	0.579*	0.706*	0.716	0.998**	0.764**	− 0.6954*	**1**

^*^Correlation is significant at the 0.05 level

^**^Correlation is significant at the 0.01 level

The association between all parameters of the roots of *H. hirsutissimum* at 20 ± 5 °C, 40 ± 5 °C, 60 ± 5 °C, and 80 ± 5 °C soil temperatures was depicted in a Pearson correlation table (Table 2). CMS showed a negative correlation with TBARS (*r* = − 0.816**) and H_2_O_2_ (*r* = − 0.643*). The root RWC also showed a strong positive correlation with proline (*r* = − 0.770**), total soluble sugar (*r* = 0.844**), and glycine betaine (*r* = 0.826**) content. The root length showed a negative correlation with TBARS (*r* = − 0.908**) and H_2_O_2_ (*r* = − 0.803**) levels (*p* < *0.01)* and with CAT (*r* = − 0.642*), APX (*r* = − 0.641*), GPX (*r* = − 0.630*) activities and AsA (*r* = − 0.706*) level (*p* < *0.05).* Moreover, antioxidant parameters were positively (*p* < *0.01*) correlated with H_2_O_2_ and TBARS levels. H_2_O_2_ level showed a strong positive correlation with CAT (*r* = 0.945**), APX (*r* = 0.964**), GPX (*r* = 0.962**) activities, and AsA (*r* = 0.977**) level.

**Table 2:** Pearson’s correlation analysis of all the parameters in the roots of *H. hirsutissimum* grown at different soil temperatures (20 ± 5 °C, 40 ± 5 °C, 60 ± 5 °C, and 80 ± 5 °C). Pro: proline, TS: total sugars, GB: glycine betaine

	Root length	RWC	TBARS	CMS	H_2_O_2_	Pro	TS	GB	CAT	APX	GPX	AsA
Root length	**1**											
RWC	− 0.487	**1**										
TBARS	− 0.908**	0.742**	**1**									
CMS	0.888**	− 0.250	− 0.816*	**1**								
H_2_O_2_	− 0.803**	0.783**	0.862**	− 0.643*	**1**							
Pro	− 0.699*	0.770**	0.743**	− 0.503	0.977**	**1**						
TS	− 0.603*	0.844**	0.677*	− 0.341	0.919**	0.969**	**1**					
GB	− 0.815**	0.826**	0.856**	− 0.582*	0.960**	0.947**	0.941**	**1**				
CAT	− 0.642*	0.809**	0.805**	− 0.566	0.945**	0.919*	0.860**	0.849**	**1**			
APX	− 0.641*	0.816**	0.740**	− 0.471	0.964**	0.984**	0.962**	0.916**	0.959**	**1**		
GPX	− 0.630*	0.819**	0.717**	− 0.423	0.962**	0.992**	0.977**	0.926**	0.934**	0.993**	**1**	
AsA	− 0.706*	0.862**	0.801**	− 0.495	0.977**	0.985**	0.976**	0.972**	0.929**	0.980**	0.986**	**1**

*Correlation is significant at the 0.05 level

**Correlation is significant at the 0.01 level

### Principal component analysis

**Fig. 10:**
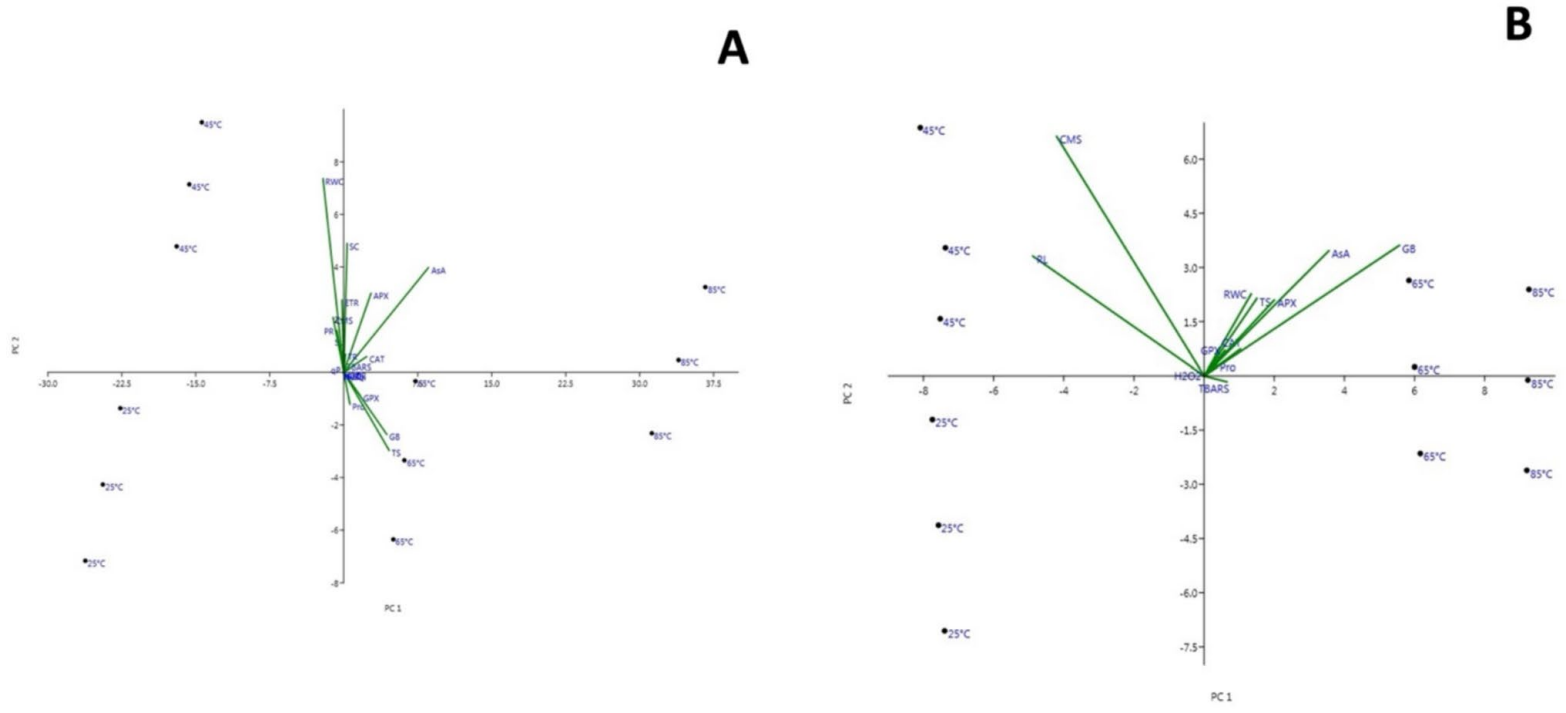
Principal component analysis (PCA) of different soil temperatures and various morphological, physiological, and biochemical variables of *H. hirsutissimum* (**A** leaves; **B** roots)*.* SL, shoot length; RT, root length; Pro, proline; TS, total sugars; GB, glycine betaine; SC, stomatal conductance; PR, photosynthetic rate; TR, transpiration rate

Principal component analysis (PCA) was performed to estimate the relative effects of leaf RWC, TBARS, H_2_O_2_, antioxidant system, gas exchange, and chlorophyll fluorescence in the leaves of *H. hirsutissimum* grown at different soil temperatures. A strong relationship was found between TBARS, H_2_O_2_, AsA, APX, CAT, GPX, and plant leaves grown at 80 ± 5 °C soil temperature. PCA showed that the increase in antioxidant capacity and osmolyte levels (proline, total soluble sugar, and glycine betaine) was higher in plant leaves grown at 60 ± 5 °C and 80 ± 5 °C. It was also determined that 40 ± 5 °C soil temperature had a high relationship with leaf RWC, ETR, and stomatal conductance. It was also found that RWC was negatively correlated with osmolyte levels, such that osmolyte accumulation increased as water content decreased (Fig. 10A).

PCA was performed to estimate the relative effects of root length, root RWC, TBARS, H_2_O_2_, and the antioxidant system in the roots of *H. hirsutissimum* grown at different soil temperatures. PCA plot revealed that plant groups grown at 20 ± 5 °C and 40 ± 5 °C are more associated with root length in particular. Analysis showed that root length was higher in plants grown at 40 ± 5 °C. It was also revealed that TBARS and H_2_O_2_ contents were lower in plant roots grown at 20 ± 5 °C and 40 ± 5 °C. At the same time, the content of AsA and the activity of APX, CAT, GPX, RWC, and osmolyte level were higher in the roots of plants grown at 60 ± 5 °C and 80 ± 5 °C; thus, the results showed that the increase in these parameters in plants grown at 60 ± 5nd 80 ± 5ontributed to heat tolerance. Finally, root RWC, AsA level, APX, CAT, and GPX activities had a strong and positive relationship with each other (Fig. 10B).

## Discussion

In our study, in order to elucidate the tolerance mechanism of *H. hirsutissimum* plant against high soil temperature, the shoot and root lengths of plants grown at different soil temperatures in the laboratory environment were determined. Then, RWC, TBARS, CMS, H_2_O_2_ analyses, osmolyte levels (proline, total soluble sugars, and glycine betaine) and some antioxidant enzyme activities were performed on the roots and leaves of the plants. Finally, changes in gas exchange parameters and chlorophyll fluorescence parameters among photosynthetic parameters were determined.

Under high temperature conditions, plants employ a wide array of survival strategies. These include both long-term evolutionary, phenological, and morphological adaptations, as well as short-term responses such as avoidance or acclimation mechanisms. Among the latter are adjustments like reorienting leaf angles, utilizing transpirational cooling, and modifying membrane lipid compositions. Typical responses to heat stress involve stomatal closure to reduce water loss, increased stomatal and trichome densities, and the development of larger xylem vessels to facilitate water transport (Caine et al. 2019). Plants under high temperature also mitigate heat stress by minimizing the absorption of solar radiation. This is often achieved through surface adaptations such as dense coverings of fine hairs and thick, waxy cuticles. These features reduce heat load and water loss. Furthermore, many of these plants exhibit leaf movements that help limit direct exposure to sunlight—for example, by orienting leaf blades parallel to incoming rays or by repositioning them during peak heat. Additionally, smaller leaves, which possess a thinner boundary layer of still air, are more efficient at dissipating excess heat, thereby conferring an advantage under elevated temperatures (Nievola et al. 2017). Our observations that *H. hirsutissimum* plants have small leaf areas and prominent hairs on leaves and stems, which are related to their ability to withstand high temperature conditions, were confirmed by the findings of Akçin et al. (2007). In addition, as mentioned before, the presence of thick cuticle in both epidermises of *H. hirsutissimum* leaves, the presence of unicellular and multicellular glandular trichomes and unicellular glandular trichomes in both epidermises, fewer stomata on the upper surface of the leaf compared to the lower surface, the upper surface of the roots being covered with multilayered periderm, the epidermal surface of the stem being covered with dense glandular and glandular hairs, and the cortex being composed of thick-walled parenchymal cells are thought to be related to tolerance to high temperatures (Akçin et al. 2007).

It is known that high temperature stress significantly affects plant growth and development by affecting shoot and root length in plants (Pregitzer et al. 2000). For this reason, in our study, firstly, the changes in shoot and root lengths with different temperature applications were determined. It was determined that shoot lengths did not differ in plants living at different soil temperatures, and root lengths decreased at high soil temperatures. It has been determined that the roots of the plants are close to the surface at high temperatures, as in their natural environment, and they do not have a deep root system. These data show that root growth of plants is more affected by temperature than shoot development. Similarly, it has been reported in the literature that the root of *H. thermophilum* decreased with increasing soil temperature (Sezgin Muslu and Kadıoğlu 2021b). It has been observed that atmospheric heat stress negatively affects the shoot lengths of 4 different genotypes of *T. aestivum* (Ahamed et al. 2010).

RWC is an important parameter that provides information about the water status of the plant. It was measured to obtain information about the water status of the plant in question under high temperature conditions. In our study, while there was no statistical difference in the relative water content in the roots of plants grown at different soil temperatures, compared to 20 ± 5 °C soil temperature, the RWC in the leaves of plants grown at 40 ± 5 °C soil temperature increased, at 60 ± 5 °C and 80 ± 5 °C. It has been found that the RWC in the leaves of plants grown at 60 ± 5 °C and 80 ± 5 °C soil temperature decreased. While it is reported in the literature that there is no significant change in the water status of tomato plants under 46 °C heat stress (Havaux 1992), in a study conducted on wheat plants, it was noted that the water status decreased under 30 °C heat stress conditions (Farooq et al. 2017).

To understand the stress status of plants under high temperature conditions, TBARS, CMS, and H_2_O_2_ contents were measured in plants under normal and high temperature conditions. The amount of TBARS is used as an indicator of the degree of membrane lipid peroxidation (Masia 2003). In our study, while there was no statistical difference in the amount of TBARS in the leaves of *H. hirsutissimum* plants under high temperature stress compared to 20 ± 5 °C soil temperature, there was no statistical difference between 40 ± 5 °C and 60 ± 5 °C soil temperature, but it increased at 80 ± 5 °C soil temperature. TBARS content in plant roots increased at high soil temperatures. Similarly, Sezgin Muslu and Kadıoğlu (2021a) noted that the amount of TBARS increased in leaves of *H. thermophilum* grown at 80 ± 5 °C, increased at 40 ± 5 °C and 60 ± 5 °C compared to that in plants at 20 ± 5 °C. Additionally, it was reported that, while the TBARS in the leaves of *H. thermophilum* increased at 55–65 °C compared to that in plants at 25–35 °C, there was no statistical difference in the TBARS in the roots of *H. thermophilum* between 25–35 °C and 55–65 °C (Bülbül et al. 2024). Cell membrane stability represents a tolerance mechanism evolved in response to membrane damage (Premachandra et al. 1991). It was revealed that cell membrane stability did not show any significant change in the roots and leaves of *H. hirsutissimum* living at high soil temperatures compared to plants at 40 ± 5 °C. Various abiotic stresses cause excessive production of reactive oxygen species that cause protein, lipid, and DNA damage in plants (Gill and Tuteja 2010). In the current study, it was determined that as soil temperature increased, H_2_O_2_ content increased in plant leaves and roots. In the literature, it was reported that the H₂O₂ content in the leaves of *H. thermophilum initially* decreased at 40 ± 5 °C with the increase in temperature and then increased at 60 ± 5 °C and 80 ± 5 °C (Sezgin Muslu and Kadıoğlu 2021b). Moreover, the H₂O₂ content in the leaves and roots of *H. thermophilum* decreased at 55–65 °C compared to that in plants at 25–35 °C (Bülbül et al. 2024). *H. hirsutissimum,* on the other hand, is capable of surviving under relatively higher heat stress conditions (80 ± 5 °C). In this context, we can also conclude that our findings align with the morphological changes (a green appearance and had vibrant leaves, flowers) observed in the plants in the present study.

Accumulation of osmolytes such as proline, glycinebetaine and trehalose is a well-known adaptation mechanism in plants to abiotic stress conditions, including temperature tolerance (Rasheed et al. 2011). It has been reported that proline and glycine betaine have functions such as osmotic adjustment, clearing ROS, protecting membrane integrity, ensuring the stability of proteins and regulating cytosolic pH, increase the accumulation of soluble sugars, and protect developing tissues from the effects of heat stress (Seki et al. 2007). According to the findings of our study, it was found that osmolytes contents increased in the leaves and roots of plants grown at high soil temperatures. Our findings suggest that the increased accumulation of osmolytes (proline, glycine betaine, and total soluble sugars) may contribute to the preservation of water status and the maintenance of turgor in *H*. *hirsutissimum* under high-temperature conditions.

Plants can adapt to oxidative stress caused by ROS accumulation and survive at high temperatures. Plant tolerance to high temperatures has been linked to increases in antioxidant enzymes such as SOD, CAT, APX, GR, and GPX (Mostofa et al. 2015). CAT is one of the most active enzymes in response to many environmental stresses, scavenging ROS (Zhang et al. 2015). H_2_O_2_ is degraded by enzymes such as CAT, APX, or GPX, resulting in a decrease in ROS accumulation (Li et al. 2020). In our study, CAT, APX, and GPX activities increased in the leaves and roots of *H. hirsutissumum* under high soil temperature. Previously, Sezgin Muslu and Kadıoğlu (2021b) reported that CAT and APX activities increased in the leaves of *H. thermophilum* with rising soil temperature under laboratory conditions. Bülbül et al. (2024) also revealed that the activities of CAT, APX, and GPX in the leaves and roots of *H. thermophilum* increased at 55–65 °C compared to those in plants at 25–35 °C. AsA, a major antioxidant in plants, tries to protect plants against oxidative stress by scavenging H_2_O_2_, etc., (Noctor et al. 2018). In the present study, AsA content in the leaves and roots of *H. hirsutissumum* markedly increased with increasing soil temperature, similar to the findings of Sezgin Muslu and Kadıoğlu (2021b), which reported that AsA content in the leaves of *H. thermophilum* increased with rising temperatures from 20 ± 5 °C to 80 ± 5 °C. The increase in CAT, APX, and GPX activities and AsA content in *H. hirsutissumum* living at high temperatures may have a pivotal role in thermotolerance by eliminating ROS (H_2_O_2_ accumulation). When the correlation results between the parameters are evaluated, the H_2_O_2_ level in plant leaves indicated a strong positive correlation with CAT (*r* = 0.892**), APX (*r* = 0.982**), GPX (0.782**) activities, and AsA (*r* = 0.951**) level (*p* < *0.01*). The H_2_O_2_ level in roots showed a strong positive correlation with CAT (*r* = 0.945**), APX (*r* = 0.964**), GPX (*r* = 0.962**) activities, and AsA (*r* = 0.977**) level (*p* < *0.01*). The current study also found that AsA content and enzyme activities in plant leaves were higher than in the roots exposed to soil temperatures at both temperature conditions. This suggests that plant roots are more adapted to high soil temperatures. Because the roots are less affected by elevated soil temperatures, they can maintain their photosynthetic activities. As a result, plants are able to survive in environments with high soil temperatures.

The reduction of photosynthesis is attributed to stomatal and non-stomatal limitations (Shangguan et al. 1999). One of these stomatal limitations is stomatal conductivity. Under high-temperature stress conditions, stomatal conductance is one of the important parameters in determining stress (Turgut and Kadıoglu 1998). In our study, it was found that stomatal conductance did not change much with high temperature. The increase of stomatal conductance results in the increase of photosynthetic capacity in leaves. The stomatal conductance decreased under 43 °C high-temperature stress conditions in nutmeg (*Jatropha curcas*) (Silva et al. 2010). The net photosynthetic rate decreases in high soil temperatures (60 ± 5 °C and 80 ± 5 °C) as opposed to those at 20 ± 5 °C and 40 ± 5 °C. In addition, it was stated that the net photosynthesis rate of the nutmeg plant decreases under heat stress conditions of 43 °C (Silva et al. 2010). The transpiration rate increased in *H. hirsutissimum* under high temperature conditions as opposed to those at 20 ± 5 °C. The transpiration data obtained in our study are in parallel with these stomatal data (Fig. 7). The correlation test also supports this situation. The stomatal conductance exhibited a positive correlation with the transpiration rate (*r* = 0.765**) (Table 1). Similarly, the transpiration rate of sorghum leaves was reported to increase under heat stress (Djanaguiraman et al. 2010). Moreover, the net photosynthesis rate of *H. thermophilum* grown at 55–65 °C was higher than that of plants grown at 25–35 °C (Bülbül et al. 2024). High stomatal conductance under elevated high soil temperature conditions indicated that CO_2_ uptake was still occurring in this study. This continuous uptake may contribute to the high net photosynthesis rate observed. Additionally, the cooling effect from evaporation played a crucial role in sustaining photosynthesis (López et al. 2022).

Photoinhibition would influence on the chlorophyll fluorescence and would inhibit ETR (Sharma and Singhal 1993). In our study, it was found that the amount of ETR increased under high temperature conditions (40 ± 5 °C and 60 ± 5 °C). The ETR in nutmeg plant decreases under 43 °C temperature stress conditions (Silva et al. 2010). Bülbül et al. (2024) also reported that the ETR of *H. thermophilum* grown at 55–65 °C was higher than that of plants grown at grown at 25–35 °C. In our study, the amount of NPQ decreased in plants under high temperature conditions compared to those at 20 ± 5 °C. Similarly, in a study on Arabidopsis mutants that the 40 °C temperature treatment resulted in a decrease in the amount of NPQ (Zhang and Sharkey 2009). In studies on wheat and Arabidopsis plants, it has been reported that the amount of NPQ in the stress situation at 45, 50, and 55 °C decreased compared to those at 40 °C, and they stated that the reason for this was that PSII activates the protective regulatory mechanism to minimize the damage (Ait Ali et al. 2008; Essemine et al. 2012; Zivcak et al. 2014). In our study, it is considered as an arrangement performed by PSII to minimize the damage and tolerate the temperature. In our study, no difference was found in the Fv/Fm ratio under high-temperature conditions compared to those at 20 ± 5 °C. Bülbül et al. (2024) determined that there was no change in the Fv/Fm ratio. In addition, Liu and Huang (2000) found that the ratio of Fv/Fm decreased under high-temperature stress conditions, while it was found that this ratio increased in cultivated creeping Bentgrass plants. This indicates that the photosynthetic membranes are protected at high temperatures. It is known that PSII is sensitive to environmental stress (Baker 1991). Various constraints, such as temperature, are thought to be the primary target in or near the reaction center of this photosystem (Kyle 1987; Lajko et al. 1997). In our study, the photochemical yield of PSII increased in plants living at 40 ± 5 °C and 60 ± 5 °C but decreased at 80 ± 5 °C compared to 20 ± 5 °C. Similarly, in the study conducted on Arabidopsis mutants, it was reported that photochemical efficiency increased under 35–42 °C heat stress conditions (Zhang and Sharkey 2009). All these data showed that under high soil temperature conditions, the heat load on the photosystems decreased, the photosynthetic machinery worked efficiently without damage, and the energy of the captured electrons was used more for photochemical reactions through ETS.

**Fig. 11:**
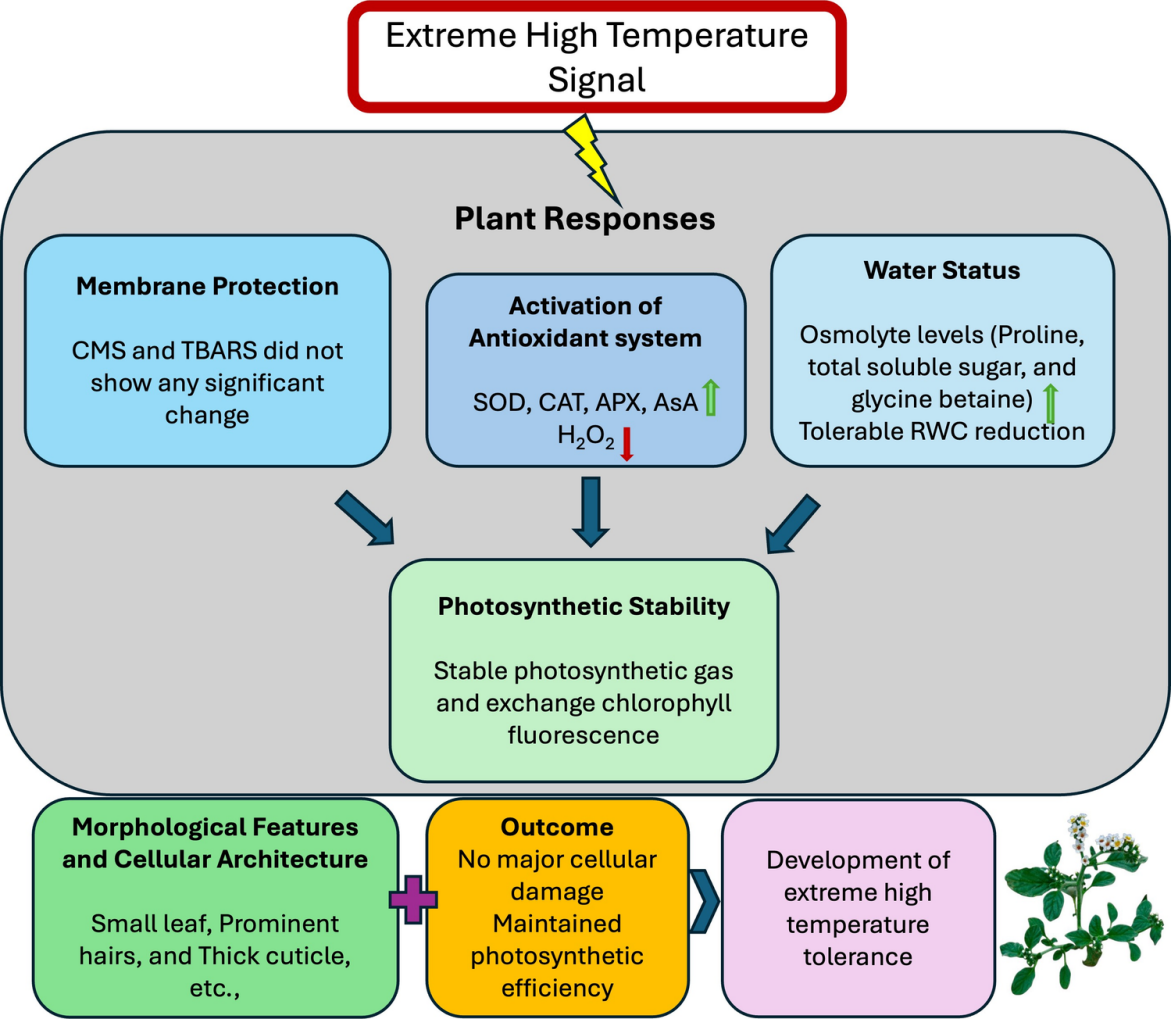
Schematic diagram showing the tolerance mechanism of *H. hirsutissimum* plant to extremely high soil temperature. CMS, cell membrane stability; TBARS, thiobarbituric acid reactive substances; RWC, relative water content; H_2_O_2_, hydrogen peroxide; SOD, superoxide dismutase; CAT, catalase; APX, ascorbate peroxidase; AsA, ascorbate

As a result, our study has shown for the first time that *H. hirsutissumum* is a high temperature tolerant plant. With the current study, the tolerance mechanism developed by *H. hirsutissumum* against extremely high temperatures was tried to be elucidated in detail at the morphological, physiological, and biochemical levels. The growth and development of *H. hirsutissumum* at four different soil temperatures (20 ± 5, 40 ± 5, 60 ± 5, and 80 ± 5 °C) were examined, and it was determined that plants continued their growth and development at all soil temperatures, including 80 ± 5 °C. Any high temperature stress damage to plant leaves that grow and develop at extremely high temperatures (80 ± 5 °C); no changes such as senescence or loss of pigment in the leaves were observed. Moreover, compared to plants at low soil temperatures, plants at high soil temperatures grew regularly with a green appearance and brilliant leaves and flowers, leading to the conclusion that high soil temperatures had no deleterious effect on them. Our findings indicated that the increase in osmolyte accumulation played a key role in sustaining water balance and protecting cell membrane integrity under high soil temperature. Moreover, the activities of some antioxidant enzymes, along with AsA content, increased under high soil temperature, and *H. hirsutissumum* effectively protects its photosynthetic units in high temperature conditions. While some stress is observed in the plant at elevated temperatures, it does not negatively impact photosynthetic efficiency. The protection of PSII function in *H. hirsutissumum* at elevated temperatures may be associated with an increase in antioxidant activities. Our results enhance the understanding of how antioxidant capacity relates to the photosynthetic performance and heat acclimation of *H. hirsutissumum*, ultimately contributing to its high temperature tolerance (Fig. 11). In conclusion, the robust antioxidant capacity of the thermotolerant *H. hirsutissumum* plays a crucial role in safeguarding its photosynthetic machinery. Research focused on enhancing the antioxidant capacity of agricultural plants under natural conditions could help protect them from heat stress and reduce the risk of decreased productivity. Moreover, with the plant’s tolerance to high temperature, for future studies, molecular research on the plant could be conducted to identify specific genes that can enhance resistance in cultivated plants.
